# Effect of frying in different culinary fats on the fatty acid composition of silver carp

**DOI:** 10.1002/fsn3.40

**Published:** 2013-05-30

**Authors:** Mahmood Naseri, Elahe Abedi, Behrooz Mohammadzadeh, Azam Afsharnaderi

**Affiliations:** 1 Department of Desert Region Management, School of Agriculture, Shiraz UniversityShiraz, Fars, Iran; 2 Department of Food science and Technology, School of Agriculture, Tarbiat Modares UniversityTehran, Iran; 3 Department of Fisheries, School of Natural Resource and Sea Science, Tarbiat Modares UniversityNoor, Mazandaran, Iran; 4 Department of Clinical Biochemistry, School of Medical Science, Tarbiat Modares UniversityTehran, Iran

**Keywords:** Fatty acid composition, frying, oil, silver carp

## Abstract

The influence of frying with four different oils (sunflower oil, soybean oil, olive oil, and corn oil) on the fatty acid composition of silver carp was evaluated. The fat content of the fillets increased after frying while the moisture content decreased in all evaluated samples. Mean saturated (SFA), monounsaturated (MUFA), polyunsaturated (PUFA) fatty acids, ∑ω_3_, and ∑ω_6_ contents of raw fish were 26.1 ± 0.5, 52.1 ± 1.1, 15.1 ± 0.6, 8.9 ± 0.1, and 6.1 ± 0.4%, respectively. Frying led to exchange of fatty acids between the silver carp lipid and frying fats. As a result of interactions, MUFA, PUFA, ∑ω_6_, and PUFA/SFA ratio of samples fried in sunflower, soybean, and corn oil significantly increased while the amounts of SFA decreased. Frying had a negative effect on the ∑ω_3_/ω_6_ ratio but reduction in olive oil-fried samples is the least among the other samples. Except in soybean oil, long-chain ω_3_-PUFA content of samples was not affected by frying.

## Introduction

Seafoods are rich sources of ω_3_-long-chain polyunsaturated fatty acids (LC-PUFA), mainly eicosapentaenoic acid (EPA, 20:5ω_3_) and docosahexaenoic acid (DHA, 22:6ω_3_). EPA and DHA appeared to play a key role in ontogenesis, especially neural development, functioning of cardiovascular system, and immune systems (Lauritzen et al. 2001). ω_3_ and ω_6_ polyunsaturated fatty acids (PUFA) are considered essential but since they cannot be synthesized in the human body, they must be obtained through diet (Mahan and Escott-Stump 2005).

Aquaculture is one of the means to achieve the nutritional goal of people in developing countries like Iran. Among the various fresh water fish supporting the Iranian fresh water fishery, carp are the most important species contributing about 58% of the total inland fish production. Silver carp (*Hypophthalmichthys molitrix*) is widely used in the composite fish culture, due to its quick growth and resistance to stress, disease, and rough handling. Consumption of this species is of crucial importance for human nutrition, especially in Iran.

The fish is commonly consumed in fried form by Iranian people. Frying, especially deep fat frying has become the most popular food preparation technology during the last six decades. The reason is that the preparation is easy even for less experienced cooks, the procedure is rapid, and the finished product is highly palatable (Gere 1982).

The complex chemical and physical changes that occur during the thermal operation result in organoleptic failures, a decrease in nutritive value, and the formation of compounds with adverse effects on health.

Studies on the fatty acid composition of silver carp usually do not take into account the influence of culinary processing, which often includes a heat treatment and fat addition. Zakipour Rahimabadi and Dad (2012) determined the influence of frying on the quality of thin slices of this species. In the mentioned research, the changes in fatty acid profiles of frying oils were not investigated. The purpose of this paper was to study how deep fat frying in different culinary fats (sunflower oil, olive oil, corn oil, and soybean oil) affected the fatty acid composition of fish fillet and frying fats, with special remarks concerning the content of EPA and DHA, LC-PUFAs and ω_3_/ω_6_ ratio changes.

## Materials and Methods

### Sample procedures

Thirty kilograms of silver carp (*H. molitrix*; 48–51 cm long and weighing 1450–1660 g) was purchased from a local store in Tehran, Iran to where they had previously been transported in refrigerated trucks from the farms of Khuzestan, Iran after catch during the winter. The lapse of time between catching and arrival at the laboratory was <30 h. Head, scale, viscera, and tail were removed, and two fillets were obtained from the resulting fish. Fillets were subsequently divided into six homogeneous groups of 2 kg each. One group was kept raw and used as the reference and the other five were fried. Frying fats were purchased from a local store in Tehran, Iran. The choice of such fats was conditioned by their fat composition: high in monounsaturated fatty acids (MUFA) in olive oil, very high in ω_6_ PUFA in sunflower oil and corn oil, and considerable amount of ω_3_ PUFA in soybean oil (Table 3).

### Frying

Silver carp fillets were fried in a deep fryer (TEFAL, Visialis trade mark, France) at 160°C for 4 min. During frying, the inner fillet temperature was monitored with an electronic thermometer (Aidin scientific trade mark, Iran). Four different vegetable oils, such as sunflower oil, olive oil, corn oil, and soybean oil, were used for frying with the food/oil ratios being 250 g/L. After frying, the fillets were gently drained for about 5 min.

### Analysis

#### Preparation of the lipid samples

All samples in each lot were homogenized using a kitchen blender and analyzed to determine moisture, total lipid, and fatty acid composition. All assays were conducted on triplicate samples of the homogenates. The moisture content of raw and fried fillets was determined by drying in an oven at 105°C until a constant weight was obtained (AOAC 1995). Lipids were extracted from the raw and fried fish flesh following the Bligh and Dyer (1959) method. Vegetable oils (sunflower oil, olive oil, corn oil, and soybean oil) before and after frying were drained with sodium sulfate and analyzed in the same way as the flesh lipid extracts.

#### Fatty acid composition

The fatty acid methyl esters (FAME) of sunflower oil, olive oil, corn oil, and soybean oil and the fish fats were analyzed by gas chromatography. FAME were prepared by methylation of the triacylglycerols, as described by Naseri et al. (2010). The FAME were analyzed using a Shimadzu 17A (Shimadzu, Kyoto, Japan) gas chromatograph equipped with a flame ionization detector and a fused silica capillary column (50–0.25°mm and 0.20 mm of Carbowax 20 mol/L). The column temperature was programmed at 2°C/min from 150 to 240°C. The injection port and detector were maintained at 220 and 245°C, respectively. The carrier gas was hydrogen (1.2 mL/min), the make-up gas was nitrogen (30 mL/min), and the split used was 1:100. The identification of normal fatty acids was done by comparing the relative retention times of FAME peaks from samples with standards from Restek (Marine Oil FAME Mix-Catalog no. 35066, Bellefonte, PA, USA) and the main fatty acids, in order of abundance, were confirmed using another Shimadzu 17A (Japan) gas chromatograph.

### Statistical analysis

SPSS, version 15.0 (Stanford, California, USA), was used for the statistical analysis. Data from the different chemical measurements were subjected to one-way analysis of variance (*P* < 0.05). Comparison of means was performed using a least-squares difference method.

## Results and Discussion

Aquatic ecosystems are known to be the main source of PUFAs and humans gain major part of EPA and DHA by consuming fish (Arts et al. 2001). Type of fish species and way of cooking (kind of heat treatment) may be important factors for content of the essential fatty acids in final products. Silver carp is an extensively cultured species. Aquaculture production of silver carp is the highest of any fish species in the world that has an annual global production of nearly 4.2 million metric tons (Naseri et al. 2010). The changes in moisture and fat content of samples after frying processes are shown in Table 1. The moisture content of the fish fillets decreased after frying while the fat content increased in all evaluated samples (*P* ≤ 0.05). According to Garcı′a-Arias et al. (2003) and Cuesta et al. (2001), during frying, oil penetrates the food after water is partially lost by evaporation. The fat content and composition of raw fish can influence fat exchanges and interactions between the culinary fat and that of the fish when frying (Sánchez-Muniz et al. 1992). The increase of the fat content in the meat fried in the PUFA-enriched culinary fat was significant and may be explained by two mechanisms, namely the loss of water during frying and the absorption of culinary fat (Hakimeh et al. 2010). These results are in agreement with those of Castrillón et al. (1997), Sánchez-Muniz et al. (1992), and Zakipour Rahimabadi and Dad (2012). According to the data of Table 1, olive oil-fried fillets had less fat than the other fried samples. Varela (1988) indicated that olive oil forms a crust that protects the food against absorption of oils, whereas other fats do not form such a defined crust and the food contains more fat after frying.

**Table 1:** Total extractable lipid and total moisture content in raw and fried silver carp

%	Raw fish	Olive oil-fried fillet	Sunflower oil-fried fillet	Corn oil-fried fillet	Soybean oil-fried fillet
Moisture	74.15 ± 0.91^a^	66.12 ± 1.31^b^	64.11 ± 1.42^c^	63.46 ± 0.89^c^	62.98 ± 1.19^c^
Lipid	10.97 ± 1.27^c^	11.85 ± 0.41^b^	13.15 ± 1.34^a^	12.98 ± 1.45^a^	13.03 ± 0.76^a^

Different letters (a>b>c) in the same row indicate a significant difference between fried samples (*P* < 0.05).

The fatty acid composition of silver carp and fried samples is shown in Table 2. The most abundant fatty acids found in raw silver carp fillets were oleic acid (C18:1ω_9_), palmitic acid (C16:0), and palmitoleic acid (C16:1ω_7_). These findings are in agreement with those obtained by Vujkovic et al. (1999). Silver carp fillets also showed considerable amounts of stearic acid (C18:0), linoleic acid (C18:2ω_6_), and DHA (C22:6ω_3_). The saturated fatty acid (SFA) content was almost double that of PUFA while the ω_3_ fatty acid content was higher than that of ω_6_ fatty acids. The amount of EPA, DHA, and PUFA in silver carp in the current study was lower than the mean values reported by Zakipour Rahimabadi and Dad (2012). However, there are differences which may depend on nutrition, sex, season, and environmental conditions.

**Table 2:** Fatty acid composition in raw and fried silver carp

Fatty acids	Raw fish	Olive oil-fried fillet	Sunflower oil-fried fillet	Corn oil-fried fillet	Soybean oil-fried fillet
14:0	1.51 ± 0.1^b^	1.61 ± 0.07^b^	2.35 ± 0.07^a^	1.47 ± 0.14^b^	1.94 ± 0.65^ab^
14:1ω_7_	0.31 ± 0. 05^b^	0.39 ± 0.03^b^	0.57 ± 0.01^a^	0.37 ± 0.02^b^	0.40 ± 0.10^ab^
16:0	20.41 ± 0.35^a^	15.65 ± 0.55^bc^	16.02 ± 0.09^b^	14.76 ± 0.69^d^	14.96 ± 0.42^cd^
16:1ω_7_	9.80 ± 0.02^b^	8.07 ± 0.56^b^	10.59 ± 0.10^a^	8.91 ± 0.55^ab^	7.16 ± 0.60^b^
18:0	2.91 ± 0.42^c^	2.75 ± 1.11^c^	2.74 ± 0.11^c^	6.63 ± 0.31^a^	4.21 ± 0.34^b^
18:1ω_9_	38.27 ± 0.84^a^	44.84 ± 3.36^a^	26.03 ± 0.44^b^	22.50 ± 1.60^bc^	20.19 ± 0.74^c^
18:1ω_7_	1.43 ± 0.23^bc^	1.21 ± 0.05^c^	1.76 ± 0.11^b^	3.88 ± 1.01^a^	3.12 ± 0.13^a^
18:2ω_6_	2.86 ± 0.11^d^	5.79 ± 0.13^c^	15.5 ± 0.09^b^	26.98 ± 4.45^a^	15.09 ± 1.11^b^
18:3ω_3_	4.87 ± 0.15^bc^	4.25 ± 0.24^c^	5.11 ± 0.02^b^	4.34 ± 0.23^bc^	7.38 ± 0.83^a^
20:1ω_11_	0.66 ± 0.03^a^	0.14 ± 0.007^bc^	0.17 ± 0.05^b^	0.15 ± 0.01^bc^	0.12 ± 0.02^c^
20:0	1.75 ± 0.06^b^	1.75 ± 0.08^b^	2.09 ± 0.02^a^	1.89 ± 0.11^ab^	1.44 ± 0.24^c^
20:2ω_6_	1.47 ± 0.06^a^	1.06 ± 0.23^b^	1.14 ± 0.02^b^	1.59 ± 0.16^a^	0.74 ± 0.12^c^
20:3ω_6_	0.51 ± 0.14^a^	0.11 ± 0.005^b^	0.19 ± 0. 01^b^	0.14 ± 0.01^b^	0.10 ± 0.06^b^
20:4ω_6_	1.35 ± 0.18^a^	0.92 ± 0.06^bc^	1.07 ± 0.05^b^	0.86 ± 0.07^bc^	0.58 ± 0.34^c^
20:5ω_3_	3.12 ± 0.42^a^	2.73 ± 0.13^ab^	3.03 ± 0.22^a^	2.52 ± 0.25^abc^	2.21 ± 0.10^c^
22:0	0.63 ± 0.14^ab^	0.69 ± 0.07^a^	0.77 ± 0.04^a^	0.67 ± 0.06^ab^	0.50 ± 0.10^b^
22:1ω_9_	0.54 ± 0.02^a^	0.52 ± 0.04^a^	0.55 ± 0.02^a^	0.51 ± 0.07^a^	0.43 ± 0.07^a^
22:6ω_3_	2.25 ± 0.05^a^	2.00 ± 0.12^a^	2.13 ± 0.18^a^	2.16 ± 0.15^a^	1.90 ± 0.38^a^
SFA^1^	26.18 ± 0.50^a^	20.87 ± 0.92^c^	22.06 ± 0.07^c^	23.69 ± 1.16^b^	21.75 ± 0.91^c^
MUFA^2^	52.14 ± 1.18^b^	56.81 ± 2.83^a^	41.87 ± 0.17^c^	38.08 ± 3.01^d^	32.78 ± 1.66^e^
PUFA^3^	15.09 ± 0.62^d^	16.89 ± 0.74^d^	28.19 ± 0.58^b^	38.63 ± 4.51^a^	28.02 ± 1.88^b^
∑ω_3_	8.90 ± 0.16^b^	8.99 ± 0.50^b^	10.28 ± 1.61^b^	9.04 ± 0.48^b^	11.50 ± 1.13^a^
∑ω_6_	6.19 ± 0.47^c^	7.89 ± 0.24^c^	17.91 ± 0.03^b^	29.59 ± 4.58^a^	16.52 ± 0.77^b^
∑ω_3_/ω_6_	1.44 ± 0.08^a^	1.14 ± 0.02^b^	0.57 ± 0.03^d^	0.32 ± 0.05^e^	0.69 ± 0.03^c^
PUFA/SFA	0.57 ± 0.02^d^	0.80 ± 0.01^c^	1.27 ± 0.02^b^	1.68 ± 0.26^a^	1.28 ± 0.03^b^

Values are percentage of total fatty acid expressed as mean ± SD of three separate determinations. Differences between letters (a>b>c) in rows indicate significant differences (*P* ≤ 0.05). SFA, saturated fatty acid; MUFA, monounsaturated fatty acid; PUFA, polyunsaturated fatty acid.

Frying in PUFA-enriched culinary fat caused a decline in the proportion of SFA, while the proportion of MUFA remained unchanged and the proportion of PUFA markedly increased in sardines (Sánchez-Muniz et al. 1992). Particularly, the proportions of linoleic acid (C18:2ω_6_) was increased because of the fatty acid (FA) composition of the fried meat that tended to become similar to that of the culinary fat (Sánchez-Muniz et al. 1992). Gall et al. (1983) found that frying with soybean oil produced a considerable decrease in the SFA content of all fish studied. Palmitic acid was the major SFA in the raw fish, but during the deep fat frying process it decreased, producing the most important reduction in sardines and mackerel. This was also found in this investigation and other researches (Gall et al. 1983; Sánchez-Muniz et al. 1992; Zakipour Rahimabadi and Dad 2012). Different effects of frying on saturated and MUFA could be explained by the kind of analyzed fish (initial fat content and fillet thickness) as well as cooking oil selected. These results are shown for silver carp under deep frying in Table 2. PUFA/SFA ratio in raw fish is 0.57 and this increased to 0.8, 1.27, 1.68, and 1.28 after frying in olive, sunflower, corn, and soybean oil due to SFA reduction and PUFA increase. These changes were not similar to those found by Zakipour Rahimabadi and Dad (2012) when olive oil was used as the medium of frying. There are differences which may depend on frying method, fillet size and thickness, quality of frying oil, and heating conditions. We observed a decline in the ratio of PUFA/SFA after frying in olive oil (Table 3).

**Table 3:** Fatty acid composition (% of total fatty acids) of frying fats before and after frying process

	Olive oil		Sunflower oil		Corn oil		Soybean oil	
				
Fatty acids	BF	AF	BF	AF	BF	AF	BF	AF
16:0	11.99 ± 0.15^b^	12.76 ± 0.32^a^	6.42 ± 0.10^a^	6.46 ± 0.01^a^	11.34 ± 0.15^a^	11.37 ± 0.13^a^	11.18 ± 0.15^a^	11.08 ± 0.30^a^
16:1ω_7_	0.72 ± 0.008	ND	ND	0.10 ± 0.05	ND	ND	ND	0.10 ± 0.007
18:0	ND	ND	4.39 ± 0.50^a^	4.42 ± 0.11^a^	2.88 ± 0.10	ND	4.09 ± 0.28^a^	3.77 ± 0.23^a^
18:1ω_9_	75.71 ± 0.85^a^	73.64 ± 1.41^a^	24.20 ± 1.31^a^	24.88 ± 0.15^a^	25.43 ± 0.75^a^	27.71 ± 2.07^a^	23.09 ± 0.96^a^	24.91 ± 0.72^a^
18:2ω_6_	9.17 ± 0.10^a^	8.83 ± 0.41^a^	62.98 ± 0.80^a^	62.20 ± 0.37^a^	57.93 ± 0.44^a^	55.10 ± 0.60^b^	53.78 ± 0.36^a^	52.05 ± 0.82^b^
18:3ω_3_	0.52 ± 0.01	ND	0.21 ± 0.03^a^	0.15 ± 0.001^a^	0.37 ± 0.01^a^	1.74 ± 0.01^a^	6.89 ± 0.04^a^	6.94 ± 0.16^a^
20:1ω_11_	0.23 ± 0.05	ND	0.14 ± 0.02^b^	0.23 ± 0.005^a^	ND	ND	ND	0.38 ± 0.008
20:0	0.40 ± 0.01^a^	0.35 ± 0.02^a^	ND	ND	ND	0.20 ± 0.04	ND	0.17 ± 0.005
20:3ω_6_	ND	0.10 ± 0.01	ND	ND	ND	ND	ND	ND
20:4ω_6_	ND	ND	ND	0.63 ± 0.005	ND	ND	ND	ND
20:5ω_3_	ND	ND	ND	0.21 ± 0.001	ND	0.15 ± 0.006	ND	0.38 ± 0.008
22:6ω_3_	ND	ND	ND	ND	ND	ND	ND	0.12 ± 0.003
∑SFA	12.40 ± 0.14^b^	13.11 ± 0.30^a^	10.81 ± 0.59^a^	10.95 ± 0.10^a^	14.22 ± 0.05^a^	11.62 ± 0.16^b^	15.27 ± 0.43^a^	15.07 ± 0.31^a^
∑MUFA	76.67 ± 0.85^a^	73.64 ± 1.41^b^	24.35 ± 1.29^a^	25.22 ± 0.15^a^	25.43 ± 0.75^a^	27.71 ± 2.07^a^	23.09 ± 0.96^b^	25.41 ± 0.73^a^
∑PUFA	9.69 ± 0.10^a^	8.94 ± 0.40^b^	63.20 ± 0.80^a^	63.20 ± 0.38^a^	58.30 ± 0.46^a^	57.007 ± 0.6^b^	60.67 ± 0.39^a^	59.51 ± 0.69^a^
∑ω_3_	0.52 ± 0.10	ND	0.21 ± 0.03^b^	0.36 ± 0.003^a^	0.37 ± 0.01^b^	1.90 ± 0.01^a^	6.89 ± 0.04^b^	7.46 ± 0.17^a^
∑ω_6_	9.17 ± 0.10^a^	8.94 ± 0.40^a^	62.98 ± 0.80^a^	62.83 ± 0.37^a^	57.93 ± 0.44^a^	55.10 ± 0.60^b^	53.78 ± 0.36^a^	52.05 ± 0.85^b^
∑ω_3_/ω_6_	0.05 ± 0.001	ND	0.003 ± 0. 005^b^	0.005 ± 0.0^a^	0.0006 ± 0.00^b^	0.03 ± 0.006^a^	0.12 ± 0.005^b^	0.14 ± 0.005^a^
PUFA/SFA	0.78 ± 0.002^a^	0.68 ± 0.03^b^	5.85 ± 0.39^a^	5.76 ± 0.08^a^	4.09 ± 0.01^b^	4.90 ± 0.02^a^	3.97 ± 0.10^a^	3.94 ± 0.11^a^

Values are percentage of total fatty acid expressed as mean ± SD of three separate determinations. Differences between letters (a>b>c) in rows indicate significant differences (*P* ≤ 0.05). BF, before frying; AF, after frying; ND, not detected; SFA, saturated fatty acid; MUFA, monounsaturated fatty acid; PUFA, polyunsaturated fatty acid.

Previous investigations showed that of the different methods of heat treatment (boiling in water, frying, roasting, grilling, oven-baking, and microwave cooking), only frying resulted in a statistically significant decrease in ω_3_ fatty acid content (García-Arias et al. 2003; Gladyshev et al. 2006). In this investigation, frying significantly changed the fatty acid composition of silver carp (Table 2). The increase in the absolute amount of ω_3_-PUFA in the used culinary fats after frying of silver carp confirms the migration of fatty acids from the silver carp. The EPA content of sunflower, soybean, and corn oil after frying increased (Table 3). Haak et al. (2007) showed that LC-PUFAs were not significantly lost by frying, but their proportions were influenced by the uptake of culinary fat. The extent of the increase or decrease of a particular FA during frying was relative to the FA gradient from the culinary fat to the fish fillet. As indicated in Tables 2 and 3, frying involves an exchange of fatty acids between the culinary fat and that of the silver carp. The interaction between both fatty products caused an increase in the silver carp muscle of proportion of the fatty acids abundant in the frying oils. Long-chain PUFA include arachidonic acid (AA, 20: 4ω_6_), EPA (20: 5ω_3_), and DHA (22: 6ω_3_). Raw silver carp has 1.35% AA that after frying in olive, sunflower, corn, and soybean oil is reduced to 0.92, 1.07, 0.86, and 0.58, respectively. Raw silver carp contains 3.12% EPA. After frying in soybean oil, the content of this fatty acid was decreased, while the other samples showed no significant change. DHA content of silver carp did not change significantly after frying. These findings are similar to those of Candella et al. (1998) and Sioen et al.(2006) in salmon. The results show that variation in DHA content is lower than that of EPA and AA. These results are in agreement with those of Echarte et al. (2001), Sioen et al. (2006), and Zakipour Rahimabadi and Dad (2012). On comparing the effects of different fats, it was discovered that frying with soybean oil is the worst way to keep the amounts of LC-PUF (AA, EPA, and DHA). Table 2 shows that the relative Σω_6_ content of silver carp increased when fried in sunflower, corn, and soybean oil. In addition importance of the amount of PUFA, ratio of ω_3_/ω_6_ is known to be of dietetic importance. According to the current WHO recommendations, ω_3_/ω_6_ PUFA should not be lower than 0.2 (Vujkovic et al. 1999). This study showed that silver carp, like other seafood, have a high ω_3_/ω_6_ ratio. Frying of silver carp accomplished with significant increasing on linoleic acid as ω_6_-PUFA, which had negative effects on the ω_3_/ω_6_ ratio. The ∑ω_3_**/**ω_6_ content in raw fish was 1.44, which then reduced to 1.14, 0.57, 0.32, and 0.69 after frying in olive, sunflower, corn, and soybean oil, respectively. ∑ω_3_**/**ω_6_ reduction in olive oil is the least among the other oils. Although the frying process decreased this ratio, the values are still higher than the recommended standard.

After frying, AA in sunflower oil, EPA in sunflower, corn and soybean oil, and finally DHA in soybean oil were detected. The ∑ω_3_**/**ω_6_ ratio increased almost in all oils after frying due to the penetration of DHA and EPA (as ω_3_ LC-PUFA) from fish lipid.

Some authors reported a decrease in PUFA levels during fish processing (Tarley et al. 2004), but it may also be species specific. Using a similar method of frying, Candella et al. (1998) and Sánchez-Muniz et al. (1992) found a threefold decrease in EPA and DHA in sardines and mackerel, whereas no significant changes in EPA and DHA content were observed (Gladyshev et al. 2006). Sebedio et al. (1993) affirmed that the longer chain ω_3_ PUFA content of mackerel was not affected by deep fat frying and geometrical fatty acid isomers of long-chain highly PUFA were formed during this process. Data published by Zakipour Rahimabadi and Dad (2012) showed that the method of frying (shallow or deep fat frying) could cause changes in the amounts of DHA and EPA. Frying in the PUFA-enriched culinary fat increased the PUFA proportion in the fish fillet but had a negative effect on the ω_3_/ω_6_ ratio.

## Conclusion

Frying led to exchange of fatty acids between the fat in the silver carp and the culinary fats used. The fat compositions of frying oils affect the fatty acid composition of silver carp. Results showed that olive oil-fried fillets had less fat than the other fried samples. The ∑ω_3_**/**ω_6_ ratio increased in all frying oils (except in olive oil) due to migration of fish fatty acids. The ∑ω_3_**/**ω_6_ content in raw fish was found to be reduced in all evaluated samples. The highest ω_3_/ω_6_ ratio was found when silver carp was fried in olive oil. Maximum reduction in EPA and DHA content was detected when soybean oil was used as the frying oil. The highest ∑ω_3_/ω_6_ content was observed in soybean oil after frying. The Major changes in ∑ω_3_/ω_6_ ratio were found in corn oil after processing.
